# Association of Breast Density with Breast Cancer Risk and Stage at Diagnosis: A Korean Nationwide Cohort Study

**DOI:** 10.3390/cancers17243897

**Published:** 2025-12-05

**Authors:** Hongki Gwak, Donghyoun Lee, Seong Hwan Kim

**Affiliations:** 1 Department of Surgery, Jeju National University Hospital, Jeju National University College of Medicine, Jeju 63241, Republic of Korea; hkgwak@jejunuh.co.kr (H.G.); dlee@jejunu.ac.kr (D.L.); 2 Department of Plastic and Reconstructive Surgery, Kangnam Sacred Heart Hospital, Hallym University College of Medicine, Seoul 07441, Republic of Korea

**Keywords:** breast cancer, breast density, diagnostic imaging

## Abstract

Background/Objectives: Breast density, as defined by the Breast Imaging Reporting and Data System (BI-RADS), reduces mammographic sensitivity and is a risk factor for breast cancer. However, its association with cancer risk and stage at diagnosis remains debated, with limited large-scale evidence. Methods: We conducted a nationwide retrospective cohort study of 952,755 Korean women who underwent screening mammography between 2013 and 2014, with breast cancer diagnoses identified over a 5-year follow-up period. Breast density was categorized by BI-RADS criteria (A–D). Breast cancer risk was evaluated using logistic regression, with odds ratios representing relative odds of developing breast cancer during the 5-year interval. Stage at diagnosis was classified as localized versus regional/distant disease according to national cancer registry records. Results: During 5 years of follow-up, 11,286 women (1.2%) were diagnosed with breast cancer. Higher breast density was significantly associated with increased breast cancer risk: multivariable-adjusted odds ratios (ORs) were 1.174 (95% CI, 1.093–1.260), 1.268 (95% CI, 1.186–1.356), and 1.287 (95% CI, 1.196–1.385) for BI-RADS B–D, respectively, compared with BI-RADS A (all *p* < 0.001). However, the risk of advanced stage (regional/distant vs. localized) disease at diagnosis did not significantly differ across breast density categories except for a modest association in BI-RADS B (OR 1.16, 95% CI, 1.01–1.33; *p* = 0.035). Conclusions: Higher breast density was independently associated with increased breast cancer risk but not with advanced-stage disease at diagnosis. These findings underscore the importance of individualized screening strategies for women with dense breasts.

## Simple Summary

Breast cancer is the most common cancer in women, and women in Asian countries are often diagnosed at an earlier age than women in Western countries. Younger women also tend to have denser breast tissue, which not only raises the risk of breast cancer but also makes it difficult for mammograms to detect tumors. It is unclear whether breast density also affects how advanced the cancer is when first diagnosed; thus, we analyzed health screening data from nearly one million Korean women to explore the link between breast density, breast cancer risk, and stage at diagnosis. We found that women with denser breasts were more likely to develop breast cancer, but their cancers were not diagnosed at a more advanced stage. These findings suggest that breast density should be considered when designing screening strategies to improve early detection.

## 1. Introduction

Breast cancer is the most common malignancy among women worldwide and a leading cause of cancer-related mortality. Although its incidence has increased globally, Asian women are typically diagnosed at younger ages compared with Western women. In Western populations, the median age at diagnosis often exceeds 60 years, whereas studies in Korea and other East Asian countries consistently report peak incidence in women in their 40s and 50s [1,2,3]. This earlier onset raises important concerns regarding screening strategies, as younger women have not only a longer life expectancy but also a higher prevalence of dense breast tissue, which is associated with both increased breast cancer risk and reduced sensitivity of mammography [4,5,6,7,8,9].

Breast density, defined by the Breast Imaging Reporting and Data System (BI-RADS), reflects the proportion of fibroglandular versus fatty tissue in the breast. High breast density decreases the sensitivity of mammography and may delay cancer detection. Moreover, it has been identified as an independent risk factor for breast cancer development [10,11,12,13,14,15]. However, whether breast density also influences stage at diagnosis remains controversial. For instance, Lynge et al. reported a substantially higher cumulative risk of breast cancer among women in the highest density category compared with those in the lowest [16], whereas Gordon et al. suggested that dense breast tissue may contribute to delayed detection and more advanced stage disease [17]. McCarthy et al. further identified breast density, prior biopsy, and family history as predictors of advanced-stage diagnosis, though results across studies have been inconsistent [18].

In Korea, BI-RADS breast density is routinely documented as part of the National Cancer Screening Program, which follows standardized quality-assurance requirements for mammographic interpretation. However, unlike regions with formal breast density notification laws, Korea does not mandate additional screening solely based on breast density. Despite high screening participation rates, large-scale Asian data evaluating both cancer risk and stage in relation to BI-RADS density remain limited.

Given these discrepancies, variations in classification criteria, and limited large-scale evidence from Asian populations, robust population-based studies using standardized definitions are required to clarify these associations. Therefore, we aimed to evaluate the relationship between BI-RADS breast density categories and both the risk of developing breast cancer and stage at diagnosis in a nationwide cohort of Korean women undergoing screening mammography. We hypothesized that higher BI-RADS breast density would be associated with an increased risk of breast cancer and a higher likelihood of diagnosis at a regional or distant stage.

## 2. Materials and Methods

### 2.1. Study Population and Data Source

This study was conducted using data from the Healthcare Big Data Integrated Platform, a national public-sector data infrastructure in Korea that provides safe and regulated access to de-identified public health and medical data. The Platform aggregates data from multiple public health institutions (including the Korea Disease Control and Prevention Agency, National Health Insurance Service, Health Insurance Review & Assessment Service, National Cancer Center, and National Medical Center), with data linkage handled via randomly generated keys issued by a trusted third party to protect personal identifiers.

Data were obtained under the platform’s application; reviewed by the research evaluation committee; and accessed in a closed, secure analytic environment. The study covered the period 2013–2014 and included individuals from the database registrant who underwent health screenings that included mammography. Incident breast cancer diagnoses were ascertained through linkage with the national cancer registry, and tumor stage at diagnosis was recorded as localized, regional, or distant. Breast cancer diagnoses were identified over a 5-year follow-up period after the baseline screening mammogram.

Breast density was classified according to the American College of Radiology (ACR) BI-RADS categories based on mammographic appearance: Category A (almost entirely fatty): breasts comprised mostly fat; Category B (scattered areas of fibroglandular density): scattered areas of density with most of the breast fatty; Category C (heterogeneously dense): more areas of fibroglandular density, which may obscure small masses; and Category D (extremely dense): breasts almost entirely dense, markedly lowering the sensitivity of mammography. Breast density was assessed from a single baseline screening mammogram interpreted by board-certified radiologists using standardized quality-assurance procedures of the National Cancer Screening Program. For women diagnosed with breast cancer, stage at diagnosis was further grouped into localized versus regional/distant disease for logistic regression analyses. Cases with unknown stage were analyzed separately or excluded in sensitivity analyses.

The primary objective of the study was to estimate the risk of breast cancer according to BI-RADS breast density categories using univariable and multivariable logistic regression, with category A serving as the reference group. The secondary objective was to investigate whether increasing breast density was associated with diagnosis at regional or distant stages compared with localized disease among women diagnosed with breast cancer. Trend analyses treating breast density as an ordinal variable were performed for both risk and stage analyses.

### 2.2. Statistical Analysis

All analyses were performed using Python 3.13.5 (Python Software Foundation, Wilmington, DE, USA). Categorical variables were summarized as counts and proportions, and comparisons between groups were initially assessed using the chi-square test.

For risk estimation, odds ratios (ORs) with 95% confidence intervals (CIs) were obtained through univariable logistic regression. Odds ratios represent the relative odds of developing breast cancer during the 5-year follow-up period. Comorbidities available in the national health screening dataset included hypertension, diabetes mellitus, dyslipidemia, heart disease, stroke, and an “other disease” category, all derived from standardized self-reported questionnaires. Among these, hypertension, diabetes mellitus, dyslipidemia, heart disease, and other chronic diseases were used as covariates in the multivariable models. Stroke was not included because the number of women reporting a prior stroke was extremely small, resulting in sparse data and unstable model convergence.

To address potential reverse causation from cancer present at the time of screening, a sensitivity analysis was conducted excluding all breast cancers diagnosed within 90 days of the index mammogram. Cox proportional hazards regression was performed with BI-RADS A as the reference category. Multivariable models were adjusted for age at diagnosis, body mass index, tumor stage (localized vs. regional/distant), hypertension, diabetes mellitus, dyslipidemia, other comorbidities, and smoking history.

Model results were visualized using forest plots, and variable contributions on the log-odds scale were further explored using SHAP-like importance measures. All statistical tests were two-sided, with *p*-values < 0.05 considered statistically significant.

## 3. Results

### 3.1. Patient Characteristics and Breast Cancer Risk

A total of 952,755 women who underwent mammography between 2013 and 2014 were included in the analysis. Women with breast cancer were more likely to be younger compared with those without breast cancer. Specifically, the incidence of breast cancer was 1.8% in women aged 40–50 years and 1.8% in those aged 50–60 years, compared with only 0.6% in women aged > 60 years (*p* < 0.001). Similarly, women with breast cancer had denser breasts, with incidence rates of 1.0%, 1.1%, 1.2%, and 1.2% across BI-RADS categories A, B, C, and D, respectively (*p* < 0.001). Higher BMI was associated with increased breast cancer risk: 1.2% in women with BMI ≥ 25 kg/m^2^ versus 1.1% in those with BMI < 25 kg/m^2^ (*p* < 0.001). In addition, women with hypertension (1.3% vs. 1.2%, *p* < 0.001) and those with a history of smoking (1.4% in current smokers, 1.2% in former smokers, vs. 1.2% in never-smokers; *p* = 0.002) had higher incidence rates compared with their counterparts (Table 1).

Univariate logistic regression showed that higher breast density was significantly associated with increased breast cancer risk. Compared with BI-RADS A, the OR was 1.11 (95% CI, 1.04–1.18; *p* = 0.002) for BI-RADS B, 1.21 (95% CI, 1.14–1.28; *p* < 0.001) for BI-RADS C, and 1.21 (95% CI, 1.14–1.30; *p* < 0.001) for BI-RADS D. Older age was associated with reduced risk (*p* < 0.001), while hypertension, higher BMI, and smoking were associated with increased risk (all *p* < 0.05). DM and dyslipidemia were not significant predictors in this analysis (Table 2).

**Figure 1:**
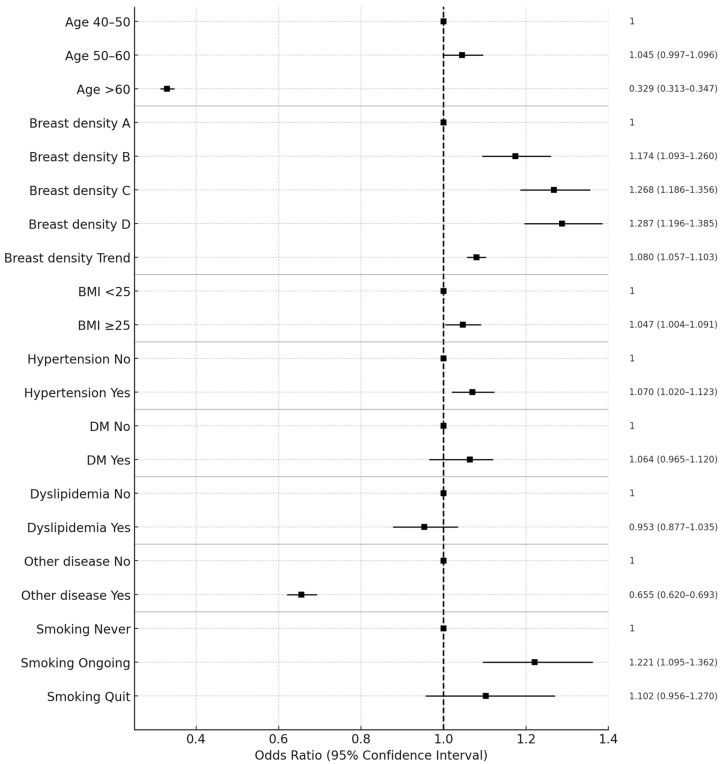
Forest plot of multivariable analysis for breast cancer risk. The vertical dashed line indicates the reference value (OR = 1.0).

Multivariable logistic regression adjusting for age, BMI, hypertension, DM, dyslipidemia, other comorbidities, and smoking history, showed that the association between breast density and breast cancer risk remained significant. Compared with BI-RADS A, the adjusted OR was 1.17 (95% CI, 1.09–1.26; *p* < 0.001) for BI-RADS B, 1.27 (95% CI, 1.19–1.36; *p* < 0.001) for BI-RADS C, and 1.29 (95% CI, 1.20–1.39; *p* < 0.001) for BI-RADS D (Figure 1). Older age remained inversely associated with breast cancer risk, whereas elevated BMI, hypertension, smoking, and other comorbidities were significant independent risk factors (all *p* < 0.05). DM and dyslipidemia were not significantly associated with breast cancer risk after adjustment (Table 3). In a sensitivity analysis excluding cancers diagnosed within 90 days of baseline screening (n = 1124 cases), the association between BI-RADS breast density and breast cancer risk remained essentially unchanged. In the restricted cohort, multivariable-adjusted odds ratios were 1.162 (95% CI, 1.081–1.249) for BI-RADS B, 1.253 (95% CI, 1.172–1.341) for BI-RADS C, and 1.276 (95% CI, 1.184–1.377) for BI-RADS D compared with BI-RADS A (all *p* < 0.001). These estimates were highly consistent with the primary results, indicating that early-detected cancers did not materially influence the overall findings.

### 3.2. Breast Cancer Stage at Diagnosis

Breast cancer stage distribution according to breast density is shown in Table 4. The proportion of patients with regional/distant disease was highest for BI-RADS B (36.3%), followed by BI-RADS C (34.9%), BI-RADS D (34.7%), and BI-RADS A (32.7%).

Multivariate logistic regression comparing regional/distant vs. localized disease revealed that only BI-RADS B showed a significantly higher risk of being diagnosed at an advanced stage compared with BI-RADS A (OR 1.16, 95% CI 1.01–1.33; *p* = 0.035). BI-RADS C (OR 1.09, 95% CI 0.96–1.25; *p* = 0.172) and BI-RADS D (OR 1.09, 95% CI 0.95–1.26 *p* = 0.229) showed no such statistical significance (Table 5). A trend analysis treating breast density as an ordinal variable showed no significant association with stage at diagnosis (OR 1.01, 95% CI 0.97–1.05; *p* = 0.708). The chi-square test across all four breast density groups also revealed no significant differences in stage distribution (*p* = 0.417).

### 3.3. Survival Analysis

In multivariable Cox regression analyses, breast density was not significantly associated with overall mortality. Compared with BI-RADS A, the adjusted hazard ratios were 0.98 (95% CI, 0.88–1.09; *p* = 0.69) for BI-RADS B, 1.02 (95% CI, 0.92–1.13; *p* = 0.71) for BI-RADS C, and 1.05 (95% CI, 0.94–1.18; *p* = 0.38) for BI-RADS D. The *p* for trend was 0.42.

## 4. Discussion

This nationwide cohort confirmed that higher breast density was independently associated with increased risk of breast cancer, consistent with findings from prior population-based studies [11,12,19,20]. Beyond reduced mammographic sensitivity, several mechanisms may contribute to this association. Dense breast tissue contains a higher proportion of epithelial and stromal components relative to adipose tissue, increasing the number of cells susceptible to malignant transformation [21]. In addition, alterations in the extracellular matrix, such as increased collagen deposition and crosslinking, have been linked to greater tissue stiffness and signaling changes that may facilitate tumor progression [22]. Genetic studies have further identified loci associated with breast density that overlap with DNA repair and cell proliferation pathways, supporting a structural and molecular basis for the observed relationship [23].

However, the influence of breast density on stage at diagnosis was limited in our cohort. Women in the BI-RADS B category showed modestly increased odds of regional or distant disease compared with those in the BI-RADS A category, while those in the BI-RADS C and D categories showed no significant differences. Prior studies have reported inconsistent findings. Gordon et al. reported that dense breast tissue may contribute to delayed detection and advanced-stage disease at diagnosis [17], and McCarthy et al. identified breast density as a risk factor for advanced-stage presentation, along with prior biopsy and family history [18]. In contrast, other population-based studies did not show a significant association after adjusting for screening practices, suggesting that, in settings with widespread access to mammography and supplemental imaging, the impact of breast density on stage at diagnosis may be attenuated [24]. In our cohort, breast density was not associated with overall mortality across BI-RADS categories, mirroring the minimal differences observed in stage at diagnosis. These findings indicate that although high breast density increases breast cancer risk, it does not appear to adversely affect prognosis once cancer has developed.

Several studies have shown that dense breast tissue is more frequently associated with estrogen receptor (ER)-positive tumors, which generally have slower progression and better prognosis compared with other subtypes [25,26,27]. If high breast density predominantly leads to ER-positive tumors, even delayed detection may not necessarily translate into a substantially higher stage at diagnosis, potentially explaining the limited stage differences observed in our cohort. Conversely, some reports have suggested associations between dense breast tissue and more aggressive subtypes [28], although results remain inconsistent. Moreover, supplemental imaging modalities such as ultrasound are frequently used in high breast density cases, improving cancer detection despite mammographic limitations [29,30,31,32]. The widespread use of these additional imaging tools likely mitigates the masking effect of breast density on stage at diagnosis and may further reduce diagnostic delays.

Although breast density has been extensively studied in Western populations, evidence from large-scale Asian cohorts remains limited. The present study strengthens the Asian dataset by providing nationwide, standardized BI-RADS data linked to cancer registry outcomes, offering a more precise estimate of both cancer risk and stage distribution across density categories. Our results indicate that, although higher breast density is a significant risk factor for breast cancer, it may not substantially delay diagnosis when supplemental imaging and organized screening programs are available. This underscores the need for risk-stratified screening strategies that integrate both cancer risk and stage information rather than focusing solely on breast density.

This study has several limitations. Its retrospective design limits the ability to establish a cause-and-effect relationship, and the use of broad stage categories (localized, regional, distant) without more detailed TNM (tumor, nodes, metastasis) classification may have masked subtle differences in disease progression. Furthermore, data on supplemental imaging modalities such as ultrasound, which are frequently used for dense breasts, were not available, limiting the assessment of their impact on cancer detection and stage at diagnosis. In addition, substantial family history information was missing and therefore could not be incorporated into the analysis.

Another important consideration is that some cancers detected shortly after the baseline screening mammogram may have already been present at the time of imaging, and therefore their stage at diagnosis may not reflect the influence of breast density. This potential misclassification could attenuate the observed association between density and stage.

Women with dense breasts are also more likely to undergo supplemental ultrasound or shorter-interval follow-up examinations, which may increase cancer detection rates and counterbalance mammographic masking. This differential screening intensity represents another source of detection bias that may partially explain the limited stage differences across density groups. As the study population consisted of women who voluntarily participated in national health screenings, selection bias cannot be excluded. These individuals may have higher health awareness and greater access to follow-up imaging, which could reduce delays in diagnosis and further diminish potential stage differences by density.

In addition, key reproductive and genetic risk factors such as parity, age at first birth, menopausal status, oral contraceptive use, hormone replacement therapy, and prior breast biopsies were not available in the dataset, limiting our ability to fully adjust for confounding in the association between density and clinical outcomes. A further limitation is that only overall survival was available in the national dataset, precluding analyses of breast cancer–specific survival or recurrence outcomes. Because overall survival incorporates non–cancer-related deaths, subtle density-related prognostic effects may have been diluted.

Despite these limitations, the study’s strengths include its large, nationwide, population-based design, the use of standardized BI-RADS breast density classification, and comprehensive adjustment for key demographic and clinical covariates. These features enhance the generalizability of our findings and provide robust evidence regarding the relationship between breast density, breast cancer risk, and stage at diagnosis. Further studies incorporating longitudinal imaging data, detailed screening histories, supplemental imaging information, and refined staging systems are warranted to better clarify how breast density influences both cancer risk and stage at presentation.

## 5. Conclusions

This large nationwide cohort study demonstrates that higher breast density is an independent risk factor for breast cancer. However, its effect on stage at diagnosis and overall prognosis appears limited in the context of contemporary screening environments where supplemental imaging is widely available. These findings highlight the importance of incorporating breast density into individualized risk-stratified screening strategies, while recognizing that density alone may not predict more advanced disease at presentation. Further studies with detailed staging, longitudinal imaging histories, and cancer-specific outcomes are warranted to refine risk assessment in women with dense breast tissue.

## Figures and Tables

**Table 1:** Patient characteristics.

	Breast Cancer (%) (*n* = 11,286)	No Cancer (%) (*n* = 941,469)	*p* Value
Age 40–50 50–60 >60	3987 (1.8) 4469 (1.8) 2830 (0.6)	223,518 (98.2) 238,288 (98.2) 479,664 (99.4)	<0.001
Breast density A B C D	1372 (1.0) 2919 (1.1) 4593 (1.2) 2402 (1.2)	131,782 (99.0) 253,778 (98.9) 365,943 (98.8) 189,967 (98.8)	<0.001
Body mass index <25 ≥25	6854 (1.1) 4432 (1.2)	589,736 (98.9) 351,734 (98.8)	<0.001
Hypertension No Yes	8655 (1.2) 2631 (1.3)	736,211 (98.8) 205,259 (98.7)	<0.001
DM No Yes	10,394 (1.2) 892 (1.2)	868,747 (98.8) 72,723 (98.8)	0.489
Heart disease No Yes	11,066 (10.7) 220 (1.1)	92,152 (89.3) 19,878 (98.9)	<0.001
Dyslipidemia No Yes	10,563 (1.2) 723 (1.2)	879,571 (98.8) 61,989 (98.8)	0.484
Other disease No Yes	9529 (1.3) 1757 (0.9)	737,653 (98.7) 203,817 (99.1)	<0.001
Smoking history Ongoing Quit Never	400 (1.4) 212 (1.2) 10,674 (1.2)	27,970 (98.6) 18,148 (98.8) 895,352 (98.8)	0.002

**Table 2:** Univariate logistic regression for breast cancer risk.

	Odds Ratio	*p* Value
Age 40–50 50–60 >60	1 1.051 (1.007–1.098) 0.331 (0.315–0.347)	0.023 <0.001
Breast density A B C D	1 1.105 (1.036–1.178) 1.206 (1.135–1.281) 1.214 (1.136–1.298)	0.002 <0.001 <0.001
Body mass index <25 ≥25	1 1.288 (1.015–13,635)	0.037
Hypertension No Yes	1 1.088 (1.039–1.140)	<0.001
DM No Yes	1 1.005 (0.940–1.074)	0.899
Heart disease No Yes	1 0.924 (0.807–1.057)	0.250
Dyslipidemia No Yes	1 0.982 (0.893–1.079)	0.720
Other disease No Yes	1 0.668 (0.624–0.714)	<0.001
Smoking history Never Ongoing Quit	1 1.245 (1.095–1.416) 1.059 (0.918–1.222)	0.001 0.446

**Table 3:** Multivariate logistic regression for breast cancer risk.

	Odds Ratio	*p* Value
Age 40–50 50–60 >60	1 1.045 (0.997–1.096) 0.329 (0.313–0.347)	0.066 <0.001
Breast density A B C D Trend	1 1.174 (1.093–1.260) 1.268 (1.186–1.356) 1.287 (1.196–1.385) 1.080 (1.057–1.103)	<0.001 <0.001 <0.001 <0.001
Body mass index <25 ≥25	1 1.047 (1.004–1.091)	0.031
Hypertension No Yes	1 1.070 (1.020–1.123)	0.005
DM No Yes	1 1.064 (0.965–1.120)	0.308
Heart disease No Yes	Not Applicable	
Dyslipidemia No Yes	1 0.953 (0.877–1.035)	0.253
Other disease No Yes	1 0.655 (0.620–0.693)	<0.001
Smoking history Never Ongoing Quit	1 1.221 (1.095–1.362) 1.102 (0.956–1.270)	<0.001 0.181

**Table 4:** Breast cancer stage at diagnosis according to breast density.

	Density A (%) (n = 1372)	Density B (n = 2919)	Density C (n = 4593)	Density D (n = 2402)	*p* Value
Local	878 (64.0)	1792 (61.4)	2873 (62.6)	1500 (62.5)	0.327
Regional	402 (29.3)	954 (32.7)	1461 (31.8)	750 (31.2)	
Distant	46 (3.4)	105 (3.6)	143 (3.1)	85 (3.5)	
Unknown	46 (3.4)	68 (2.3)	116 (2.5)	67 (2.8)	

**Table 5:** Multivariable analysis of breast density and stage at diagnosis.

	OR (Local vs. Regional + Distant)	*p* Value
Density A	1	
Density B	1.158 (1.010–1.328)	0.035
Density C	1.094 (0.962–1.245)	0.172
Density D	1.091 (0.947–1.257)	0.229
Trend	1.008 (0.967–1.051	0.708

## Data Availability

The data presented in this study are available in the National Health Insurance Service (NHIS) repository under license. Information on data access procedures is available at the NHIS Big Data Portal: https://nhiss.nhis.or.kr (accessed on 30 November 2025).

## Abbreviations

The following abbreviations are used in this manuscript:

BI-RADS	Breast Imaging Reporting and Data System
TNM	Tumor, Nodes, Metastasis
ER	Estrogen receptor
BMI	Body mass index
DM	Diabetes mellitus
OR	Odds ratio
CI	Confidence interval
